# Are Early Relapses in Advanced-Stage Ovarian Cancer Doomed to a Poor Prognosis?

**DOI:** 10.1371/journal.pone.0147787

**Published:** 2016-01-28

**Authors:** Fabien Vidal, Paul Guerby, Mathieu Luyckx, Pascale Haddad, Eberhard Stoeckle, Philippe Morice, Eric Leblanc, Fabrice Lecuru, Emile Daraï, Jean Marc Classe, Christophe Pomel, Thomas Filleron, Gwenael Ferron, Denis Querleu, Arash Rafii

**Affiliations:** 1 Stem cell and microenvironment laboratory, Weill Cornell Medical College in Qatar, Education City, Qatar Foundation, Doha, Qatar; 2 Department of Genetic Medicine, Weill Cornell Medical College, New York, New York, United States of America; 3 Department of Gynecologic Surgery, Toulouse Academic Hospital, F-31059, Toulouse, France; 4 Department of Gynecologic Surgery, Saint Luc Academic Hospital, Catholic University of Louvain, Bruxelles, Belgium; 5 Biostatistics Core, Weill Cornell Medical College in Qatar, Education City, Qatar Foundation, Doha, Qatar; 6 Department of Surgery, Institut Bergonie, Comprehensive Cancer Center, Bordeaux, France; 7 Department of Gynecologic Surgery, Institut Gustave Roussy, Cancer Campus, Grand Paris, Villejuif, France; 8 Department of Gynecologic Oncology, Centre Oscar Lambret, F-59037, Lille, France; 9 Department of Gynecologic Oncology, Georges Pompidou European Hospital, Paris, France; 10 Department of Gynecologic Surgery, Tenon Hospital, Paris, France; 11 Department of Surgical Oncology, Centre Gauducheau, Comprehensive Cancer Center, Saint Herblain, France; 12 Department of Surgical Oncology, Jean Perrin Cancer Center, Clermont-Ferrand, France; 13 Department of Surgical Oncology, Institut Claudius Regaud, Comprehensive Cancer Center, F-31052 Toulouse, France; Indiana University School of Medicine, UNITED STATES

## Abstract

**Objective:**

Early recurrence (ER) after completion of therapeutic regimen in advanced-stage ovarian cancer is a challenging clinical situation. Patients are perceived as invariably having a poor prognosis. We investigated the possibility of defining different prognostic subgroups and the parameters implicated in prognosis of ER patients.

**Study Design:**

We analyzed a multi-centric database of 527 FIGO stage IIIC and IV ovarian cancer patients. We defined patients relapsing within 12 months as ER and investigated using Cox logistic regression the prognostic factors in ER group. We subsequently divided ER patients into good and poor prognosis groups according to a lower or higher overall survival (OS) at 12 months after relapse and determined parameters associated to poor prognosis.

**Results:**

The median follow up was 49 months. One hundred and thirty eight patients recurred within 12 months. OS and Disease Free Survival (DFS) were 24.6 and 8.6 months, respectively, in this group of patients. Among the ER patients, 73 had a poor prognosis with an OS after relapse below 12 months (mean OS = 5.2 months) and 65 survived after one year (mean OS = 26.9 months). Residual disease (RD) after debulking surgery and mucinous histological subtype negatively impacted prognosis (HR = 1.758, *p* = 0.017 and HR = 8.641, *p = 0*.*001* respectively). The relative risk of death within 12 months following relapse in ER patients was 1.61 according to RD status. However, RD did not affect DFS (HR = 0.889, *p = 0*.*5*).

**Conclusion:**

ER in advanced-stage ovarian cancer does not inevitably portend a short-term poor prognosis. RD status after initial cytoreduction strongly modulates OS, that gives additional support to the concept of maximum surgical effort even in patients who will experience early recurrence. The heterogeneity in outcomes within the ER group suggests a role for tumor biology in addition to classical clinical parameters.

## Introduction

Early relapse in cancer management is still a challenging clinical situation. Ovarian carcinoma is a locally metastatic disease at presentation (FIGO stages III and IV), as cancer spreads into the abdominal cavity before symptoms occur [1, 2]. The mainstay of treatment involves whenever possible complete cytoreductive surgery associated with platinum and taxane-based chemotherapy [3–5]. However, despite achievement of complete clinical remission after initial treatment, 60% of patients with advanced stages will relapse within five years [6]. Among them, those presenting with early recurrence are often perceived as having a poor prognosis. To date, the choice of second line therapeutic agents is based on interval to relapse [7]. Therefore, platinum-based combination therapy is usually recommended in patients with recurrence from 6 to 12 months after completion of first line treatment while diseases recurring within the first 6 months are considered platinum resistant and require different therapeutic regimen [8–10].

Nevertheless, early recurrence in ovarian cancer as a clinical entity is not well defined yet. Thereby, specific research focusing on clinical characteristics and outcomes in such patients is lacking, beyond therapeutic considerations. Thus, we aim to define the characteristics of advanced-stage diseases relapsing within 12 months after completion of initial treatment, and to identify different prognostic subgroups.

## Patients and Methods

### Study design

We analyzed a database of 527 patients presenting with advanced-stage ovarian, tubal or peritoneal epithelial carcinoma (FIGO IIIC and IV with pleural invasion only) treated in 7 French gynecologic oncology units from January 2003 to December 2007 [11]. All patients received optimal first line treatment involving a combination of platinium and taxane-based chemotherapy and curative debulking surgery. Toulouse, Paris, Villejuif, Bordeaux, Nantes, Lille and Clermont-Ferrand institutional review boards granted permission for this retrospective and observational study. Patients’ records and information were anonymized and de-identified prior to data analysis. No written consent was given by the patients for their inclusion in the study.

We stratified the population according to disease-free survival (DFS). Patients who relapsed within the first 12 months constituted the early relapse group (ER); patients relapsing after 12 months were included into the late relapse group (LR). No relapse group (NR) corresponded to patients showing no evidence of recurrence after at least 36 months of follow-up. We excluded all the patients without relapse whose follow-up was less than 36 months. We then divided ER patients into good prognosis (GPER) and poor prognosis (PPER) groups according to overall survival (OS) after relapse, with a cut-off at 12 months. Recurrence was systematically assessed by conventional imaging (computed tomography), PET scan or laparoscopic exploration. Therefore, isolated subsequent increase in CA 125 level was not defined as a relapse.

### Disease characteristics

Peritoneal carcinomatosis was quantified using the peritoneal index cancer (PCI) [12] and the extent of upper abdominal disease. Surgical procedures were sorted into 3 categories, according to the extent of resection [11]. Group 1 included standard procedures with hysterectomy, salpingo-ophorectomy, rectosigmoid resection, infra-gastric omentectomy, pelvic and para-aortic lymph node dissection and appendicectomy. Group 2 comprised all radical debulking surgeries. Group 2A patients underwent standard surgery plus routine upper abdominal procedure (stripping of diaphragmatic peritoneum, splenectomy). Group 2B consisted in ultra-radical surgeries involving a combination of digestive tract resections, organ resection (spleen, bladder, stomach), coeliac lymphadenectomy and total abdominal peritoneum stripping, in addition to standard surgery. Regarding residual disease, patients were classified into 2 groups: no visible residual disease, and visible residual disease.

### Statistical analysis

Statistical analysis was performed using XLSTAT (Addinsoft^**®**^, USA). OS and disease-free survival (DFS) were computed as previously described [11]. The first-event corresponded to death of any cause for OS and to relapse or cancer-related death for DFS. OS and DFS curves were achieved using Kaplan Meier analysis. The Cox proportional hazard regression model was used for multivariate analysis. All statistical tests were 2 sided and differences were considered statistically significant when *p< 0*.*05*.

## Results

### Characteristics of patients with early recurrence

This previously described database was consistent regarding demographics, therapeutic management and outcomes with previous studies focusing on advanced-stage ovarian cancer. The median follow up time was 49 months. Complete cytoreduction with no tumor residue was achieved in 374 patients (71%). Upfront surgery was performed in 190 patients (36%). Mean DFS was 28.3 months and survival rate was 54% at 48 months [11].

Among our study population, 138 patients (26.2%) recurred within 12 months following primary treatment (ER group) and 275 (52.2%) after 12 months (LR group). One hundred fourteen patients (21.6%) did not develop any recurrence but only 68 had a follow-up of at least 36 months and were finally included (NR group). Comparative demographics are displayed in Table 1. Patients with ER had more poor prognostic factors. PCI, residual disease and stage IV rate were significantly higher than in the other groups. Noteworthy, no difference was found between ER and LR regarding treatment schedule and extent of surgery (standard or radical procedures). ER patients had a poorer outcome with a significant decrease in OS compared to LR patients (24.6 versus 60.9 months, respectively; *p<0*.*001*). Similarly, OS after recurrence was significantly shorter in patients with ER than in those with LR (16.1 versus 37 months, respectively; *p<0*.*001*).

**Table 1:** Comparative demographics between Early Relapse, Late Relapse and No Relapse groups.

	Early Relapse *n = 138*	Late relapse *n = 275*	No relapse *n = 68*	*p* value
**Age**, mean (SD)	59 (10.8)	58 (10.7)	59 (10.0)	*0*.*30*
**PCI**, mean (SD)	13 (7.0)^A^	11 (6.7)^B^	8 (5.0)^B^	*0*.001*
**Stage**	^A^	^B^	^C^	*0*.03*
IIIC	106 (76.8%)	233 (84.7%)	62 (91.2%)	
IV	32 (23.2%)	42 (15.3%)	6 (8.8%)	
**Neo-adjuvant CT**	^A^^B^	^A^	^B^	*0*.02*
Yes	89 (64.5%)	191 (69.5%)	37 (54.4%)	
No	49 (35.5%)	84 (30.5%)	31 (45.6%)	
**Histological type**	^A^	^B^	^A^^-^^B^	*0*.004*
Serous papillary	91 (65.9%)	214 (77.8%)	42 (61.9%)	
Endometrioid	19 (13.8%)	20 (7.3%)	10 (14.7%)	
Clear cells	8 (5.8%)	3 (1.1%)	2 (2.9%)	
Undifferentiated	11 (8.0%)	29 (10.5%)	9 (13.2%)	
Mucinous	5 (3.6%)	3 (1.1%)	3 (4.4%)	
Other	4 (2.9%)	6 (2.2%)	2 (2.9%)	
**Grade**	^A^	^A^^-^^B^	^B^	*0*.01*
1	5 (3.6%)	21 (7.6%)	6 (8.8%)	
2	44 (31.9%)	71 (25.8%)	10 (14.7%)	
3	63 (45.6%)	115 (41.8%)	39 (57.3%)	
**Residual disease**	^A^	^B^	^C^	*0*.006*
No	79 (57.3%)	197 (71.6%)	61 (89.7%)	
Yes	59 (25.7%)	77 (28%)	7 (10.3%)	
*1-10mm*	*34*	*53*	*3*	
*> 10mm*	*25*	*24*	*4*	
**Surgical extent**				*<0*.001*
1	65 (47.1%)^A^	147 (53.5%)^A^	54 (79.4%)^B^	
2A	34 (24.6%)	57 (20.7%)	10 (14.7%)	
2B	39 (28.3%)	71 (25.8%)	4 (5.9%)	
**Courses of CT**, mean (SD)	6.6 (3.8)	7.7 (3.8)	6.2 (3.3)	*0*.*13*

^A-B-C^: there is a statistical significance in the comparison between the groups marked with a different letter.

SD = Standard deviation; PCI = Peritoneal Cancer Index; CT = chemotherapy.

### Prognostic factors within the ER group

We performed a Cox logistic regression to determine the prognostic factors in ER group (Tables 2 and 3). On bivariate analysis, mucinous histological subtype and grade 1 were associated with decreased OS (HR of 4.788 and 2.912, respectively) while endometrioid subtypes yielded a better prognosis (HR = 0.530). After multivariate analysis, mucinous subtype was the only biological feature that negatively impacted prognosis (HR = 8.641, *p = 0*.*001*).

**Table 2:** Cox logistic regression in Early Relapse group: bivariate analysis.

	Overall survival
	HR IC (95%) *p*
**Residual disease**	
No	1
Yes	1.678 1.15–2.45 *0*.008*
**Surgical extent**	
Standard	1
(ultra) Radical	0.776 0.53–1.14 *0*.*20*
**Stage**	
IIIC	1
IV	0.910 0.58–1.42 *0*.*68*
**Neoadjuvant CT**	
Yes	1
No	1.294 0.88–1.90 *0*.*19*
**Histological type**	
Papillary serous	1
Endometrioid	0.530 0.29–0.98 *0*.04*
Undifferentiated	0.715 0.33–1.56 *0*.*40*
Mucinous	4.788 1.89–12.12 *0*.001*
Clear cells	0.708 0.30–1.62 *0*.*41*
Others	1.753 0.63–4.84 *0*.*28*
**Grade**	
3	1
2	1.140 0.74–1.75 *0*.*55*
1	2.912 1.136–7.464 *0*.03*

CT = chemotherapy.

**Table 3:** Cox logistic regression in Early Relapse group: multivariate analysis.

	B	SE	Wald	df	*p-*value	HR
**Residual disease** (Yes)	0.564	0.235	5.743	1	*0*.02*	**1.758**
**Surgical extent** (Radical)	-0.364	0.247	2.176	1	*0*.*14*	0.695
**Stage** (IV)	-0.408	0.288	2.006	1	*0*.*16*	0.665
**Histological type**			15.985	5	*0*.007*	
Endometrioid	-0.697	0.356	3.838	1	*0*.05*	**0.498**
Undifferentiated	-0.164	0.428	0.146	1	*0*.*70*	0.849
Mucinous	2.156	0.645	11.171	1	*0*.001*	**8.641**
Clear cells	-0.351	0.511	0.473	1	*0*.*49*	0.704
Others	0.620	0.638	0.943	1	*0*.*33*	1.858
**Neoadjuvant CT** (Yes)	-0.132	0.230	0.328	1	*0*.*57*	0.876
**Grade**			2.762	2	*0*.*25*	
1	0.827	0.504	2.688	1	*0*.*10*	2.285
2	0.035	0.241	0.021	1	*0*.*88*	1.035

CT = chemotherapy.

B = Beta (maximum likelihood estimation); SE = standard error; df = degrees of freedom; HR = hazard ratio.

**Fig 1:**
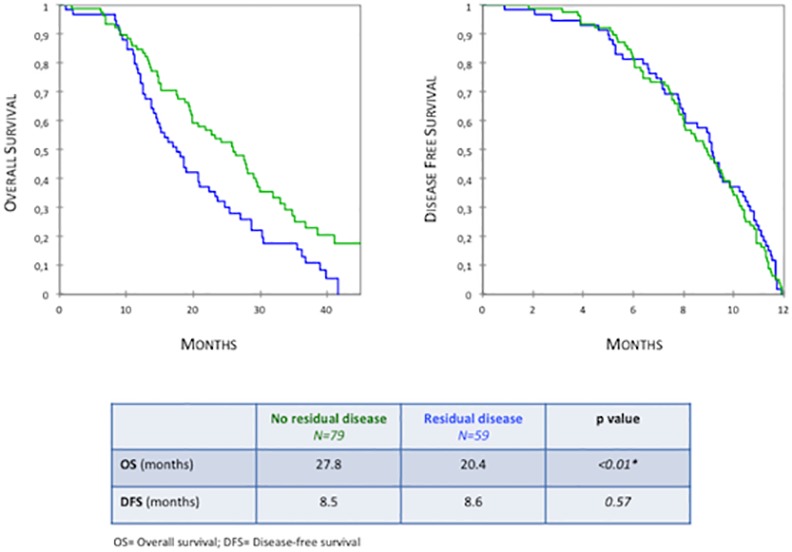
Overall Survival and Disease-Free Survival in patients with early relapse according to residual disease status (Kaplan Meier analysis).

Residual disease after debulking surgery was the only clinical factor influencing OS on both bivariate and multivariate analyses (HR = 1.758, *p = 0*.*02*). Patients with no residual disease had significantly improved OS compared to patients with residual disease (27.8 versus 20.4 months, respectively; *p = 0*.*007*) (Fig 1). They also displayed increased OS after recurrence (19.3 versus 11.9 months, respectively; *p = 0*.*008*). Interestingly, the presence of tumor residue at the end of surgery did not impact DFS (HR = 0.889, *p = 0*.*5*). Concordantly, Kaplan Meier analysis did not show any difference in DFS according to residual disease status (8.5 months in the absence of residual tumor versus 8.6 months; *p = 0*.*64*) (Fig 1).

Within the ER population, complete cytoreduction was achieved in 79 women (57.2%). In these patients, mean PCI was 12 (+/- 7.7) and did not significantly differ neither from the whole cohort of ER patients (13 +/- 7, *p = 0*.*33*) nor from the subgroup with residual disease (15 +/- 6, *p = 0*.*07*) (S1 Table). There was no statistical difference in treatment schedule between ER patients with complete resection and those with residual disease after surgery: rates of neo-adjuvant chemotherapy were respectively 68.3% and 59.3% (*p = 0*.*27*). We did not observe any significant difference in recurrence sites and types (isolated or multiple) according to residual disease status, whereas we were expecting more peritoneal relapses in patients with tumor residues.

**Fig 2:**
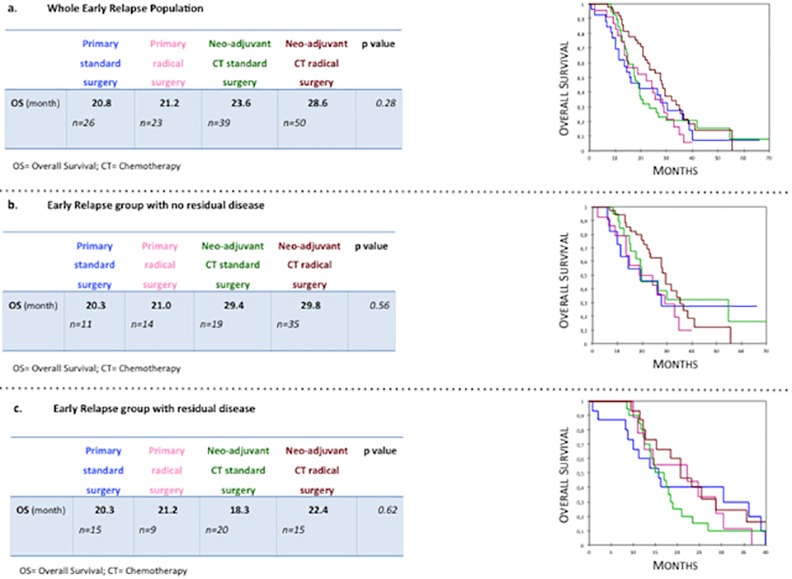
Comparative outcomes according to treatment patterns in (a) whole Early Relapse population, (b) patients with complete and (c) incomplete cytoreductive surgery (Kaplan Meier analysis).

To determine if therapeutic modalities impacted prognosis, we compared the survival outcomes associated with the following patterns of treatment: (1) upfront standard surgery, (2) upfront radical surgery, (3) neoadjuvant chemotherapy followed by standard surgery and (4) neoadjuvant chemotherapy followed by radical surgery. We did not observe any significant difference in OS between all subgroups (Fig 2).

### Inter-patients heterogeneity in the ER group

Among the ER group, 73 patients died within the first year following their relapse and constituted the poor prognosis (PPER) group. The good prognosis (GPER) group comprised 65 women whose OS after recurrence was greater than 12 months. OS after recurrence was 5.2 (+/- 3.6) months in PPER patients and 26.9 (+/- 9.8) months in GPER group. There were no differences between the 2 groups regarding stage, PCI, histological type and patterns of treatment and recurrence (Table 4). Interestingly, both groups had similar DFS (9 months for GPER versus 8.2 for PPER, *p = 0*.*12*). Absence of residual disease was correlated to a better prognosis and was achieved in a greater proportion of GPER patients (*p = 0*.*02*). The relative risk of death within 12 months following recurrence in ER patients was 1.61 according to residual disease status. The matching hazard ratio was 1.70 (CI 1.07–2.69, *p = 0*.*02*).

**Table 4:** Comparative characteristics between poor prognosis and good prognosis Early Relapse patients.

	Poor prognosis *n = 73*	Good prognosis *n = 65*	*p* value
**Age**, mean (SD)	61 (11.6)	58 (9.7)	*0*.*08*
**Upper abdominal disease**			*0*.*41*
No	20	12	
Yes	40	42	
*≤25mm*	*25*	*26*	
*>25mm*	*15*	*16*	
**PCI**, mean (SD)	12 (7.2)	14 (6.7)	*0*.*28*
**Stage**			*0*.*44*
IIIC	58 (79.5%)	48 (73.8%)	
IV	15 (20.5%)	17 (26.2%)	
**Neo adjuvant CT**			*0*.*70*
Yes	46 (63.0%)	43 (66.2%)	
No	27 (37.0%)	22 (33.8%)	
**Histological type**			*0*.*11*
Papillary serous	51 (69.9%)	40 (61.5%)	
Endometrioid	6 (8.2%)	13 (20.0%)	
Undifferentiated	3 (4.1%)	5 (7.7%)	
Mucinous	6 (8.2%)	5 (7.7%)	
Clear cells	5 (6.9%)	0 (0.0%)	
Others	2 (2.7%)	2 (3.1%)	
**Grade**			*0*.*08*
1	5 (6.8%)	0 (0.0%)	
2	23 (31.5%)	21 (32.3%)	
3	31 (42.5%)	33 (50.8%)	
**Residual disease**			*0*.02*
No	35 (47.9%) ^A^	44 (67.7%) ^B^	
Yes	38 (52.1%)	21 (32.3%)	
*1-10mm*	*21*	*13*	
*> 10mm*	*17*	*8*	
**Surgical extent**			*0*.02*
1	41 (56.2%) ^A^	24 (36.9%) ^B^	
2	32 (43.8%)	41 (63.1%)	
*2A*	*14*	*20*	
*2B*	*18*	*21*	
**DFS** (month)	8.2	9.0	*0*.*12*
**Patterns of recurrence**			*0*.*83*
Peritoneum	48	44	
Lymph node	14	11	
Lung	4	5	
Liver	10	6	
Multiple	21	14	
**Courses of CT**, mean (SD)	6.7 (4.0)	6.6 (3.5)	*0*.*92*

^A-B^: there is a statistical significance in the comparison between the groups marked with a different letter.

SD = Standard deviation; NS = non significant; PCI = Peritoneal Cancer Index; CT = chemotherapy.

Focusing on patients with no residual disease after surgery, there were no significant differences in clinical parameters between the PPER and GPER groups (S2 Table). PPER patients without tumor residue had a poorer OS compared to the pool of ER patients with residual disease with a gap of 7.3 months (13.1 versus 20.4 months, respectively; *p = 0*.*006*). Conversely, we found no difference in OS among PPER population according to residual disease status (13.1 months in the absence of residual disease versus 13.5 months; *p = 0*.*88*).

Despite the identification of inter-patient heterogeneity in prognosis among ER patients, the overall survivals associated with GPER group did not overlap with those observed in the LR patients: overall survival and OS after recurrence were significantly shorter in the GPER group (37.4 months versus 61.6, *p*<10^−3^ for OS; 28.5 months versus 37.3, *p* = 0.023 regarding OS after relapse).

### Intercenter comparison

In all centers, treatment modalities and schedule were defined in tumor review board and based on French and International guidelines. Maximal surgical effort was performed to achieve complete cytoreduction with no tumor residue whenever applicable.

Mean DFS and OS in the database population were 28.3 months and 57.8 months respectively. Survival outcomes were homogeneous between the centers, except for one department that displayed increased OS (66.7 months, *p*<10^−3^) and DFS (35.3 months, *p* = 0.032). Focusing on ER subgroups, no differences were found between the 7 centers in OS and OS after recurrence (*p* = 0.223 and *p* = 0.219, respectively).

Overall disease recurred in 413 patients along the study period. The global rate of ER was thus 33.4%. Intercenter comparison did not find any difference in the occurrence of ER (*p* = 0.68).

## Discussion

Our study demonstrates that early relapses in advanced-stage ovarian cancer are not all doomed to a poor prognosis, as we have identified 2 subgroups with distinct survival profiles. Within the early relapse (ER) patients, the absence of residual disease after surgery is the most important clinical prognostic factor. This adds to current knowledge, as this supports a “biological” effectiveness of complete surgery, even when the underlying biological characteristics of the tumor are unfavorable.

In the era of personalized precision medicine it is quite important to determine major clinical prognostic factors that will leverage our use of biomarkers. Early recurrence is perceived as a major factor of poor prognosis and treatment regimen is at this point chosen based on the timing of relapse rather than other considerations. Early relapses are usually considered as those occurring within 6 months after completion of first line treatment. However, we consider this definition to be restrictive, mainly because clinical and radiological diagnosis may be delayed relative to the pathologic reality of the recurrence. Our aim was to focus on spontaneous prognosis after recurrence, regardless of second line treatments, and our analysis revealed no significant difference in outcomes after recurrence occurring within the first 6 or 12 months. Therefore, we considered as early every relapse arising in the first year of follow-up.

The prognostic impact of surgical debulking in advanced ovarian cancer is well known. Nowadays, complete resection has replaced the former concept of optimal cytoreduction and requires trained teams and multidisciplinary approaches [3, 5, 13–15]. Du Bois *et al* have confirmed in their combined exploratory analysis that the absence of residual disease after surgery results in better outcomes for both OS and DFS, compared to incomplete cytoreduction [16].

Concordantly, complete resection with no tumor residue led to better OS and DFS in our multi-centric setting [11]. Focusing on the ER group, residual disease was the only significant clinical factor impacting OS. Surprisingly, it did not affect DFS and sites of recurrence. Further hypotheses ensue from such findings. (1) Complete cytoreduction may have somehow a beneficial effect on the course of disease sensitivity to second line treatments in ER patients, without influencing recurrence kinetics and patterns. Similar observation had been only mentioned twice without a focus on early relapses [16, 17]. Unfortunately, response rate weighted by residual disease after primary debulking surgery is often lacking in trials assessing second line treatments. (2) Beyond surgical considerations, tumor biology may determine disease resectability and overall sensitivity to first and second line treatments. For instance, the subgroup of poor prognosis patients (PPER) with complete cytoreduction displayed poorer outcomes than the pool of ER women who underwent incomplete debulking surgeries, with a gap of 7.3 months in OS. Moreover, residual disease status did not impact survival in PPER patients. Although it is based on a limited number of patients, such finding suggests that biological factors may portend a stronger impact on disease outcomes than the completeness of tumoral resection and take part in survival heterogeneity. In our database, we have found that mucinous histological subtype was associated with poorer outcomes (HR = 8.641, *p = 0*.*001*), as previously reported [18]. Identification of additional biological features would allow the clinicians to prevent patients from undergoing labor intensive and unnecessary morbid cytoreductive procedures.

To date, it remains unclear how achieving initial complete resection positively impacts on prognosis after an early relapse. Actually, it is equally uncertain that we should consider the “positive impact” of complete cytoreduction rather than the “negative impact” of incomplete debulking. In ovarian cancer, specific data are lacking about surgical stress feed back on cancer cells plasticity and response to chemotherapy. In breast cancer, incomplete surgical resection of tumor is responsible for ERBB2 overexpression in cancer cells, resulting in stimulation of growth and poorer prognosis [19, 20]. Increased secretion of inflammatory cytokines such as IL6 and IL8 has been observed following intra-abdominal surgical stress [21]. Autocrine production of IL6 and IL8 confers cisplatin and paclitaxel resistance in ovarian cancer cells, due to increased expression of both multidrug resistance-related genes and apoptosis inhibitory proteins [22–24]. Then, may the inverse approach be true? Instead of always considering that prognosis is mostly supported by the absence of residual disease, we should reconsider the negative impact of incomplete cytoreduction that might increase chemo-resistance of residual disease [25].

Our results also reveal the complexity of ovarian cancer outcome above common consideration and suggests considering treatment pattern for personalized medicine rather than treatment regimen. Indeed some patients will probably achieve longer survival undergoing primarily radical surgeries while others may benefit from more a stepwise approach of neoadjuvant therapy associated with standard surgery if this allows complete surgery. The biology of the peritoneum and tumor might be determinant in stratifying patients.

We have used the concept of “overall survival after recurrence” to determine the diversity in prognosis within the group of patients with early relapse. Defining a cut-off at 12 months, we have uncovered large inter-patients heterogeneity: the gap in OS after relapse was 21.7 months between PPER and GPER patients. While our study does have limitations (cut-off arbitrarily defined, retrospective design, missing data regarding second line treatments), such heterogeneity has several consequences. We must reconsider our clinical attitude based on the DFS and try to consider other factors to optimize second line therapy. We must design early relapse specific trials that will uncover the biological factors predicting poor prognosis.

In conclusion, ER in advanced-stage ovarian cancer does not inevitably lead to a short-term poor prognosis since we have identified subgroups displaying different outcomes. The amount of residual disease left after initial debulking surgery does not seem to impact on disease-free interval but strongly modulates OS. Our analysis set the ground for a change in attitude toward patients with early relapse, as platinum resistance might not actually entirely correlate with timeframe. The lack of major improvement in ovarian cancer patients outcome might then be due to a lack of perception of the humongous heterogeneity of this disease amplified by the presentation at metastatic stage. Future trial should consider both clinical as well as biological features to optimize patients’ therapy.

## Supporting Information

S1 Table Comparative demographics in Early Relapse patients according to residual disease status. (DOCX)

S2 Table Comparative demographics in Early Relapse patients with complete cytoreductive surgery according to prognosis group. (DOCX)
