# Supplementary material for: Are Early Relapses in Advanced-Stage Ovarian Cancer Doomed to a Poor Prognosis?
Source: PLoS One. 2016 Jan 28;11(1):e0147787. doi: 10.1371/journal.pone.0147787 (PMC4731146; doi:10.1371/journal.pone.0147787)
Supplement: S1 Table — (DOCX) [file pone.0147787.s001.docx]

**S1 table.** Comparative demographics in Early Relapse patients according to residual disease status

|  | No residual disease  *n=79* | Residual disease  *n= 59* | *p* value |
| --- | --- | --- | --- |
| **Age**, mean (SD) | 59 (10.8) | 63 (9.7) | *0.06* |
| **PCI**, mean (SD) | 12(7.7) | 15 (6.0) | *0.07* |
| **Stage** |  |  | *0.08* |
| IIIC | 65 (82.3%) | 41 (69.5%) |  |
| IV | 14 (17.7%) | 18 (30.5%) |  |
| **Neo-adjuvant CT** |  |  | *0.27* |
| Yes | 54 (68.3%) | 35 (59.3%) |  |
| No | 25 (21.7%) | 24 (40.7%) |  |
| **Surgical extent** |  |  | *0.01** |
| 1 | 30 (38.0%) ^A^ | 35 (59.3%) ^B^ |  |
| 2 | 49 (62.0%) | 24 (40.7%) |  |
| *2A* | *21* | *13* |  |
| *2B* | *28* | *11* |  |
| **Patterns of recurrence** |  |  | *0.85* |
| Peritoneum | 57 | 35 |  |
| Lymph node | 14 | 11 |  |
| Lung | 5 | 4 |  |
| Liver | 11 | 5 |  |
| Multiple | 23 | 12 |  |
| **Courses of CT**, mean (SD) | 6.8 (3.5) | 6.3 (4.0) | *0.39* |

^A-B^: there is a statistical significance in the comparison between the groups marked with a different letter

SD= Standard deviation; PCI= Peritoneal Cancer Index; CT= chemotherapy
