# Supplementary material for: Are Early Relapses in Advanced-Stage Ovarian Cancer Doomed to a Poor Prognosis?
Source: PLoS One. 2016 Jan 28;11(1):e0147787. doi: 10.1371/journal.pone.0147787 (PMC4731146; doi:10.1371/journal.pone.0147787)
Supplement: S2 Table — (DOCX) [file pone.0147787.s002.docx]

**S2 table.**Comparative demographics in Early Relapse patients with complete cytoreductive surgery according to prognosis group

|  | Poor prognosis  *n=35* | Good prognosis  *n=44* | *p* value |
| --- | --- | --- | --- |
| **Age**, mean (SD) | 58 (13.0) | 57 (10.0) | *0.71* |
| **Upper abdominal disease** |  |  | *0.24* |
| No | 19 | 18 |  |
| Yes | 16 | 26 |  |
| *< 2.5cm* | *9* | *15* |  |
| *> 2.5cm* | *7* | *11* |  |
| **PCI**, mean (SD) | 10 (8.0) | 13 (7.1) | *0.19* |
| **Stage** |  |  | *0.47* |
| IIIC | 30 (85.7%) | 35 (79.5%) |  |
| IV | 5 (14.3%) | 9 (20.5%) |  |
| **Neo adjuvant CT** |  |  | *0.35* |
| Yes | 22 (63.0%) | 32 (72.7%) |  |
| No | 13 (37.0%) | 12 (27.3%) |  |
| **Histological type** |  |  | *0.06* |
| Serous papillary | 22 (63.0%) | 28 (63.6%) |  |
| Endometrioid | 2 (5.7%) | 8 (18.2%) |  |
| Clear cells | 2 (5.7%) | 3 (6.8%) |  |
| Undifferentiated | 4 (11.4%) | 3 (6.8%) |  |
| Mucinous | 4 (11.4%) | - |  |
| Other | 1 (2.8%) | 2 (4.6%) |  |
| **Surgical extent** |  |  | *0.21* |
| 1 | 16 (45.7%) | 14 (31.8%) |  |
| 2 | 19 (54.3%) | 30 (68.2%) |  |
| *2A* | *8* | *13* |  |
| *2B* | *11* | *17* |  |
| **DFS** (month) | 7.96 | 8.98 | *0.19* |
| **Patterns of recurrence** |  |  | *0.43* |
| Peritoneum | 25 | 32 |  |
| Lymph node | 6 | 8 |  |
| Lung | 4 | 1 |  |
| Liver | 6 | 5 |  |
| Multiple | 13 | 10 |  |

SD= Standard deviation; PCI= Peritoneal Cancer Index; CT= chemotherapy
